# Bioactive Constituents Obtained from the Seeds of *Lepidium apetalum* Willd

**DOI:** 10.3390/molecules22040540

**Published:** 2017-03-28

**Authors:** Sijian Wang, Pingping Shi, Lu Qu, Jingya Ruan, Shengcai Yang, Haiyang Yu, Yi Zhang, Tao Wang

**Affiliations:** 1 Tianjin State Key Laboratory of Modern Chinese Medicine, 312 Anshanxi Road, Nankai District, Tianjin 300193, China; 15122587883@163.com (S.W.); qululuhan88@163.com (L.Q.); Ruanjy19930919@163.com (J.R.); yuhaiyang19830116@hotmail.com (H.Y.); 2 Tianjin Key Laboratory of TCM Chemistry and Analysis, Institute of Traditional Chinese Medicine, Tianjin University of Traditional Chinese Medicine, 312 Anshanxi Road, Nankai District, Tianjin 300193, China; shipingpingtcm@163.com (P.S.); 15122473723@163.com (S.Y.)

**Keywords:** *Lepidium apetalum*, flavonoid glycosides, phenolic glycosides, HepG2 cells, triglyceride accumulation inhibitory effects

## Abstract

Three new compounds, apetalumosides C_1_ (**1**), D (**2**), and 1-thio-β-d-glucopyranosyl(1→1)-1-thio-α-d-glucopyranoside (**3**), together with twenty-two known ones (**4**–**25**) were obtained from the seeds of *Lepidium apetalum* Willd. Among the known isolates, **5**–**8**, **10**–**13**, **16**–**20**, and **25** were obtained from the genus for the first time; **4**, **14**, **15**, and **21**–**24** were isolated from the species for the first time. Meanwhile, the NMR data of **16** was first reported here. Their structures were determined by means of chemical and spectroscopic methods. On the other hand, their inhibitory effects on sodium oleate-induced triglyceride (TG) overloading in HepG2 cells were evaluated. As a result, two new compounds (**1** and **2**), together with known isolates **7**–**11**, **13**, **14**, **16**–**18**, **20**, **21**, and **25** possessed significant inhibitory effects in the cells.

## 1. Introduction

In our on-going program of screening the phytochemical and bioactive constituents from *Lepidium apetalum* seed extract [1,2], three new compounds, apetalumosides C_1_ (**1**), D (**2**), and 1-thio-β-d-glucopyranosyl(1→1)-1-thio-α-d-glucopyranoside (**3**), along with twenty-two known isolates, astragalin (**4**) [3]; kaempferol 3-*O*-β-d-glucopyranosyl-7-*O*-β-d-gentiobioside (**5**) [4]; drabanemoroside (**6**) [5]; quercetin 3-*O*-β-d-glucopyranosyl-7-*O*-β-d-gentiobioside (**7**) [4]; quercetin 3-*O*-α-l-rhamnopyranosyl(1→2)-α-l-arabinopyranoside (**8**) [6]; isorhamnetin 3-*O*-β-d-glucopyranoside (**9**) [7]; isorhamnetin 3,4′-*O*-β-d-diglucoside (**10**) [8]; isorhamnetin 3-*O*-β-d-glucopyranosyl-7-*O*-β-d-gentiobioside (**11**) [4]; 2-*O*-(3,4-dihydroxybenzoyl)-2,4,6-trihydroxyphenylacetic acid 4-*O*-β-d-glucopyranoside (**12**) [9]; 4,9-di-*O*-β-d-glucosyl sinapoyl alcohol (**13**) [10]; 3′,5′-dimethoxy-4-*O*-β-d-glucopyranosyl cinnamic acid (**14**) [11]; sinapoylglucose (**15**) [12]; sinapoyl-9-sucrosecoside (**16**); 1(*E*),2(*E*)-di-*O*-sinapoyl-β-d-glucopyranoside (**17**) [13]; 1,2-disinapoylgentiobiose (**18**) [14]; lariciresinol 4′-*O*-β-d-glucopyranoside (**19**) [15,16]; (7*S*,8*R*)-aegineoside (**20**) [17,18]; l-tryptophan (**21**) [19]; thymidine (**22**) [20]; adenosine (**23**) [21]; stachyose (**24**) [22]; and TgSSTg (**25**) [23] were obtained. Among the known isolates, **5**–**8**, **10**–**13**, **16**–**20**, and **25** were obtained from the genus for the first time. Meanwhile, **4**, **14**, **15**, and **21**–**24** were isolated from the species for the first time, and the NMR data of **16** was first reported here. Moreover, as the active ingredients of the hypolipidemic effect, several phenolic compounds, including five flavonoids (**7**–**11**), five sinapic acid homologues (**13**, **14**, and **16**–**18**), and one lignan (**20**), together with two new compounds (**1** and **2**), as well as two other isolates (**21** and **25**) exhibited significant triglyceride (TG)-lowering effects in HepG2 cells.

## 2. Results and Discussion

**Figure 1:**
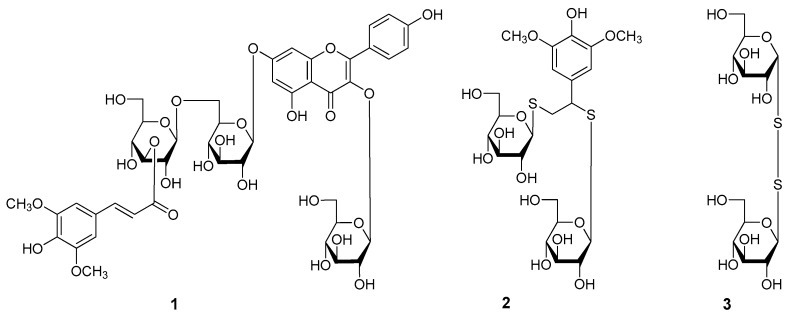
The new compounds **1**–**3** obtained from the seeds of *L. apetalum*.

**Figure 2:**
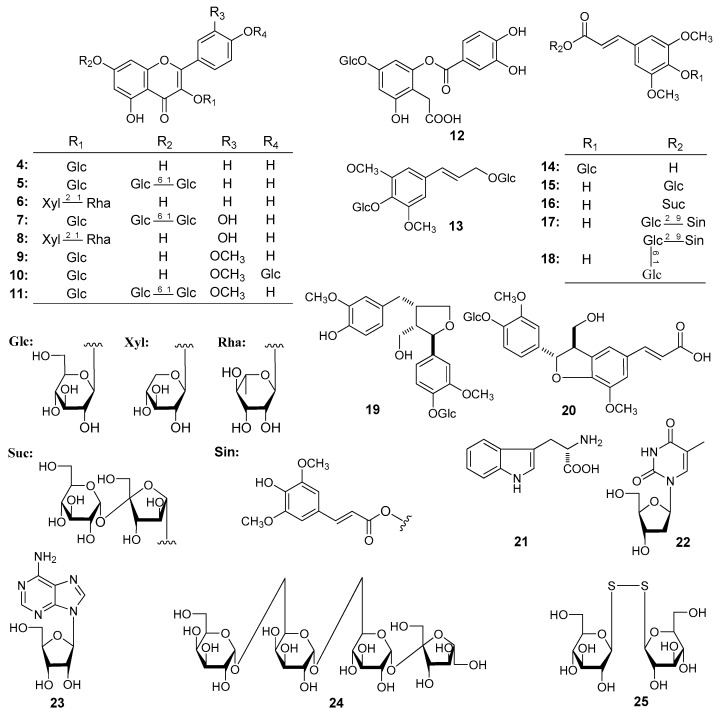
The known compounds (**4**–**25**) obtained from the seeds of *L. apetalum*.

The 50% EtOH extract of *L. apetalum* seeds was treated with the same experimental process as reported in reference [1,2] to obtain 95% EtOH eluate, which was separated by silica gel, octadecylsilica (ODS), Sephadex LH-20 CC, and finally preparative HPLC to yield compounds **1**–**25**. Their structures are shown in Figure 1 and Figure 2.

*Apetalumoside C_1_* (**1**) was isolated as yellow powder with negative optical rotation ([*α*]${}_{D}^{25}$ −41.1°, MeOH). Its molecular formula was deduced as C_44_H_50_O_25_ from a [M − H]^−^ quasi-molecular ion at *m*/*z* 977.2555 (calcd. for C_44_H_49_O_25_, 977.2568) in the negative-ion HRESI–TOF–MS spectrum. The ^1^H-, ^13^C-NMR (Table 1) and 2D NMR (^1^H-^1^H COSY, HSQC, HMBC, HSQC–TOCSY) spectra revealed the occurrence of one kaempferol aglycon (δ 6.51 (1H, br. s, H-6), 6.85 (1H, br. s, H-8), 6.92 (2H, d, *J* = 9.0 Hz, H-3′,5′), 8.09 (2H, d, *J* = 9.0 Hz, H-2′,6′), 12.65 (1H, br. s, 5-OH)); three β-d-glucopyranosyl (δ 4.35 (1H, d, *J* = 8.0 Hz, H-1′′′′), 5.12 (1H, d, *J* = 7.0 Hz, H-1′′′), 5.50 (1H, d, *J* = 8.0 Hz, H-1′′)); along with one sinapoyl (δ_H_ 3.81 (6H, s, 3′′′′′,5′′′′′-OCH_3_), 6.53 (1H, d, *J* = 16.0 Hz, H-8′′′′′), 7.00 (2H, s, H-2′′′′′,6′′′′′), 7.51 (1H, d, *J* = 16.0 Hz, H-7′′′′′); δ_C_ 166.2 (C-9′′′′′)). Meanwhile, in the HMBC experiment, the long-range correlations from H-1′′ to C-3; H-1′′′ to C-7; H-1′′′′ to C-6′′′; H-3′′′′ to C-9′′′′′ were observed, then the connectivities between oligoglycoside moieties and aglycon or sinapoyl groups were characterized. Finally, a HSQC–TOCSY experiment was developed to assign the badly overlapped protons in the sugar chemical shift range. In the HSQC–TOCSY spectrum, correlations between the following proton and carbon pairs were observed: δ_C_ 100.6 (C-1′′) and δ_H_ 3.08 (H-4′′), 3.21 (H-2′′), 3.26 (H-3′′), 5.50 (H-1′′); δ_H_ 3.08 (H-4′′) and δ_C_ 60.8 (C-6′′), 69.8 (C-4′′), 74.2 (C-2′′), 76.3 (C-5′′), 76.8 (C-3′′); δ_H_ 5.12 (H-1′′′) and δ_C_ 69.2 (C-4′′′), 73.0 (C-2′′′), 76.2 (C-3′′′), 99.7 (C-1′′′); δ_H_ 3.71, 3.99 (H_2_-6′′′) and δ_C_ 68.9 (C-6′′′), 69.2 (C-4′′′), 73.0 (C-2′′′), 75.3 (C-5′′′), 76.2 (C-3′′′); δ_C_ 103.5 (C-1′′′′) and δ_H_ 3.22 (H-2′′′′), 3.34 (H-4′′′′), 4.35 (H-1′′′′), 4.90 (H-3′′′′); δ_H_ 4.90 (H-3′′′′) and δ_C_ 60.7 (C-6′′′′), 68.1 (C-4′′′′), 77.4 (C-5′′′′), 103.5 (C-1′′′′). Acid hydrolysis of **1** yielded d-glucose, which was identified by retention time and optical rotation using chiral detection by HPLC analysis [1,2].

**Figure 3:**
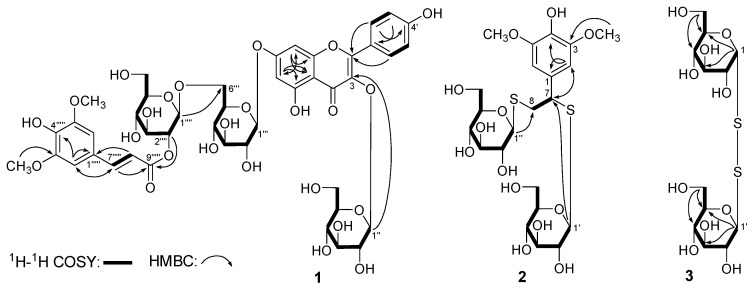
The main ^1^H-^1^H COSY and HMBC correlations of **1**–**3**.

*Apetalumoside D* (**2**), white powder, exhibited negative optical rotation ([*α*]${}_{D}^{25}$ −35.3°, in MeOH). In the positive-ion HRESI–TOF–MS of **2**, the quasi-molecular ion peak was observed at *m*/*z* 593.1333 [M + Na]^+^ (calcd. for C_22_H_34_O_13_S_2_Na, 593.1333), and its molecular formula was revealed to be C_22_H_34_O_13_S_2_. The ^1^H-, ^13^C-NMR spectra (Table 2) indicated the presences of one symmetrical 1,3,4,5-tetrasubstituted benzene ring (δ 6.58 (2H, s, H-2,6)); two methoxyl (δ 3.75 (6H, s, 3,5-OCH_3_)); one oxygenated methene (δ 3.18 (1H, br. d, ca. *J* = 11 Hz), 3.39 (1H, dd, *J* = 5.0, 11.0 Hz), H_2_-8); one methine bearing an oxygen function (δ 4.28 (1H, br. d, ca. *J* = 5 Hz, H-7)); along with two 1-thio-β-d-glucopyranosyl (δ 4.27 (1H, d, *J* = 10.0 Hz, H-1′′), 4.31 (1H, d, *J* = 9.5 Hz, H-1′)) [24]. The ^1^H-^1^H COSY experiment on **2** indicated the presence of three partial structures shown in bold bonds (Figure 3). Finally, the planar structure of apetalumoside D (**2**) was determined by the long-range correlations from H-2,6 to C-1, 3–5, 7; 3,5-OCH_3_ to C-3,5; H-7 to C-1, 2,6, 8, C-1′; H-1′ to C-7; H-1′′ to C-8 observed in its HMBC spectrum. The ^1^H- and ^13^C-NMR data of **2** was assigned by the correlations from proton to carbon displayed in the HSQC spectrum.

The molecular formula of 1-thio-β-d-glucopyranosyl(1→1)-1-thio-α-d-glucopyranoside (**3**) was deduced as C_12_H_22_O_10_S_2_ from a [M + H]^+^ quasi-molecular ion at *m*/*z* 391.0739 (calcd. for C_12_H_23_O_10_S_2_, 391.0727). Twelve signals were displayed in its ^13^C-NMR (Table 3) spectrum, and all of their chemical shifts appeared in the field of 60–100. The correlations from δ_H_ 4.68 (1H, d, *J* = 9.0 Hz, H-1′) to δ_C_ 92.4 (C-1′), and δ_H_ 5.57 (1H, d, *J* = 5.5 Hz, H-1) to δ_C_ 96.1 (C-1) observed in the HSQC spectrum indicated that there were two sugar units in **3**. Combined with its MS and ^1^H-NMR spectrum (Table 3), the presence of two 1-thio-glucopyranosyl parts were conjectured. Among them, the anomeric proton (δ_H_ 4.68 (H-1′)) and a set of ^13^C-NMR (δ_C_ 63.7 (C-6′), 72.1 (C-4′), 74.4 (C-2′), 80.0 (C-3′), 83.1 (C-5′), 92.4 (C-1′)) signals revealed the presence of 1-thio-β-d-glucopyranosyl [23,24]. Meanwhile, the presence of 1-thio-α-d-glucopyranosyl was presumed by the following signals: δ_H_ 5.57 (H-1), and δ_C_ 63.4 (C-6), 72.3 (C-4), 74.3 (C-2), 76.2 (C-3), 76.4 (C-5), 96.1 (C-1). Moreover, all of the coupling constants between H-2 and H-3, H-3 and H-4, H-4 and H-5 were 9.5 Hz, which indicated that the protons in C-2, 3, 4, 5 were in axial bond. On the other hand, H-1 was suggested to be in equatorial bond by *J*_H-1,2_ = 5.5 Hz. Finally, the nuclear overhauser effect (NOE) correlations between H-2 and H-1, H-4; H-3 and H-5 observed in the NOESY experiment, further proved the presence of 1-thio-α-d-glucopyranosyl. The assignment of protons and carbons was reached by the ^1^H-^1^H COSY, HSQC, and HMBC spectra. On the basis of the above mentioned evidence, the structure of **3** was elucidated to be 1-thio-β-d-glucopyranosyl(1→1)-1-thio-α-d-glucopyranoside (**3**).

**Figure 4:**
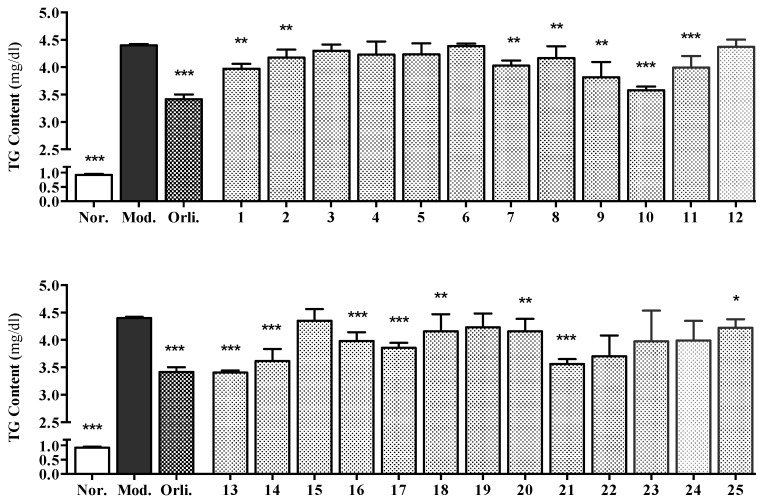
Effects of compounds **1**–**25** on TG overloading in HepG2 cells. Cells were treated with 200 µmol/L sodium oleate (SO) for 48 h. Meanwhile, 30 µmol/L-tested compounds or 5 µmol/L-orlistat (Orli.) were co-incubated to evaluate their inhibitory effects, respectively. Each value represents the mean ± S.E.M., *n* = 4, *** *p* < 0.001, ** *p* < 0.01, * *p* < 0.05 vs. model group (Mod.). Nor. = normal group.

The *L. apetalum* isolates were evaluated for their inhibitory activities on TG overloading by the model of sodium oleate (SO)-induced fatty liver in vitro. As shown in Figure 4, compounds **1**, **2**, **7**–**10**, **11**, **13**, **14**, **16**–**18**, **20**, **21** and **25** exhibited significant TG-lowering effects, among which, **10**, **13** and **21** showed levels of activities almost equivalent to the positive control—a TG clearance rate of about 22%—and the remainders also reached at least 4.02% ± 1.57%.

**Figure 5:**
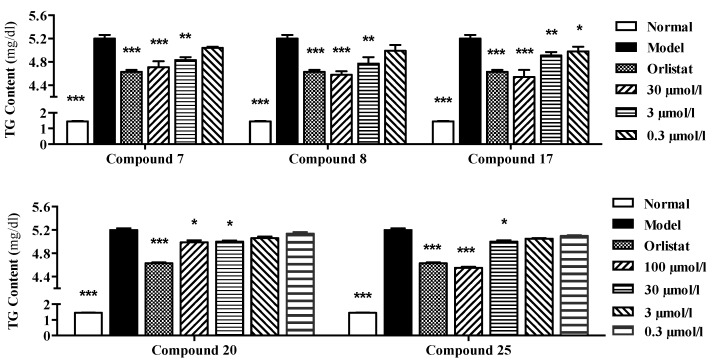
Concentration-dependent inhibitory effects of compounds **7**, **8**, **17**, **20**, and **25** on TG overloading in HepG2 cells. Cells were treated with 200 µmol/L SO for 48 h. Meanwhile, different indicated concentrations of tested compounds were co-incubated to perform the dose dependency study, respectively. Each value represents the mean ± S.E.M., *n* = 4, *** *p* < 0.001, ** *p* < 0.01, * *p* < 0.05 vs. model group (Mod.). Nor. = normal group.

According to the results shown in Figure 5, the tested compounds **7**, **8**, **17**, **20** and **25** showed different dose–activity relationships. In response to stimulations of **7**, **8** and **17** (at 30, 3 and 0.3 µmol/L), or **20** and **25** (at 100, 30, 3 and 0.3 µmol/L), gradual decrement trends of TG overloading were observed (shown in Table 4 and Table 5).

As for structure–activity relationships, quercetin glycosides (**7** and **8**) and isorhamnetin glycosides (**9**–**11**) in the current study showed significant TG-lowering effects, while kaempferol glycosides (**4**–**6**) exhibited no obvious activity, which indicated that the 3′-position substitution of hydroxyl or methoxy might play critical roles on the TG-lowering activity of flavone glycosides. For apetalumoside C1 (**1**), a previous study has reported that the substitution of 7-position by *O*-glycosides would reduce the inhibitory activities of flavonoid glycosides [2], while in the current study, **1** still exhibited a strong effect with the glycosylation of 7-hydroxyl; this is speculated to be due to the presence of the sinapoyl group in the structure. Meanwhile, five of the six sinapic acid homologues in our study, including **13**, **14**, and **16**–**18**, showed significant TG-lowering activities. By comparing the TG clearance rate of **17** (12.39% ± 0.95%) with that of **18** (5.49% ± 3.17%), at the concentration of 30 µmol/L, as well as the difference of their structures, we speculated that the one additional glycosyl might be the reason for the reduced activity. However, it is noteworthy that sinapoylglucose (**15**) showed lower activity than that of sinapoyl-9-sucrosecoside (**16**), which made it complicated to illustrate the influence of the substituted position and amount of glycosyl on the activity of sinapic acid groups.

## 3. Experimental

### 3.1. General

Ultraviolet–visible spectroscopy (UV) and Infrared Spectroscopy (IR) spectra were recorded on a Varian Cary 50 UV-Vis (Varian Australia Pty Ltd., Mulgrave, Australia) and Varian 640-IR FT-IR spectrophotometer (Varian, Inc., Hubbardsdon, MA, USA), respectively. Optical rotations were measured on a Rudolph Autopol IV automatic polarimeter (Rudolph Research Analytical, Hackettstown, NJ, USA). NMR spectra were determined on a Bruker 500 MHz NMR spectrometer (Bruker BioSpin AG Industriestrasse, Fällanden, Switzerland) at 500 MHz for ^1^H- and 125 MHz for ^13^C-NMR (internal standard: tetramethylsilane). Negative- and positive-ion mode HRESI–TOF–MS were obtained on an Agilent Technologies 6520 Accurate-Mass Q-TOF LC/MS spectrometer (Agilent Corp., Santa Clara, CA, USA).

Column chromatographies (CC) were performed on macroporous resin D101 (Haiguang Chemical Co., Ltd., Tianjin, China), silica gel (48–75 μm, Qingdao Haiyang Chemical Co., Ltd., Qingdao, China), ODS (40–63 μm, YMC Co., Ltd., Tokyo, Japan), and Sephadex LH-20 (Ge Healthcare Bio-Sciences, Uppsala, Sweden). Preparative high performance liquid chromatography (PHPLC) columns, Cosmosil 5C_18_-MS-II (20 mm i.d. × 250 mm, Nakalai Tesque, Inc., Tokyo, Japan), were used to separate the constituents.

### 3.2. Plant Material

The seeds of *L. apetalum* were collected from Anguo city, China, and identified by Dr. Li Tianxiang (The Hall of Traditional Chinese Medicines (TCM) Specimens, Tianjin University of TCM, Tianjin, China). The voucher specimen was deposited at the Academy of Traditional Chinese Medicine of Tianjin University of TCM (No. 20120501).

### 3.3. Extraction and Isolation

The seeds of *L. apetalum* (10 kg) were treated with the same experimental process as reported in reference [1,2], as a result, the 95% EtOH (Fraction 1) and H_2_O (Fraction 2) eluates were obtained.

Fraction 1 (80 g) was subjected to silica gel CC (CHCl_3_–MeOH (100:0 → 100:5, *v*/*v*) → CHCl_3_–MeOH–H_2_O (10:3:1 → 6:4:1, lower layer, *v*/*v*) → MeOH) to yield sixteen fractions (Fr. 1-1–1-16). Fractions 1-7 (12.5 g) and 1-8 (12.0 g) were isolated by ODS CC (MeOH–H_2_O (20% → 30% → 40% → 50% → 70% → 100%, *v*/*v*)); as a result, fifteen (Fr. 1-7-1–1-7-15) and eleven fractions (Fr. 1-8-1–1-8-11) were obtained, respectively. Fraction 1-7-1 (699.0 mg) was prepared by PHPLC [CH_3_CN–H_2_O (5:95, *v*/*v*) + 1% HOAc] to give thymidine (**22**, 11.1 mg). Fraction 1-8-1 (253.1 mg) was purified by PHPLC [CH_3_CN–H_2_O (8:92, *v*/*v*) + 1% HOAc] to yield 3′,5′-dimethoxy-4-*O*-β-d-glucopyranosyl cinnamic acid (**14**, 29.7 mg). Fraction 1-8-3 (579.2 mg) was separated by PHPLC (CH_3_CN–H_2_O (1:99, *v*/*v*) + 1% HOAc), and adenosine (**23**, 22.7 mg) was gained. Fraction 1-8-4 (1.3 g) was isolated by PHPLC (CH_3_CN–H_2_O (9:91, *v*/*v*) + 1% HOAc) to yield seven fractions (Fr. 1-8-4-1–1-8-4-7). Fraction 1-8-4-5 (130.9 mg) was further purified by PHPLC (MeOH–H_2_O (22:78, *v*/*v*) + 1% HOAc) to yield sinapoylglucose (**15**, 7.3 mg). Fraction 1-8-7 (573.6 mg) was separated by Sephadex LH-20 CC (MeOH–H_2_O (1:1, *v*/*v*)) and PHPLC (CH_3_CN–H_2_O (15:85, *v*/*v*) + 1% HOAc) to give lariciresinol 4′-*O*-β-d-glucopyranoside (**19**, 6.5 mg). Fraction 1-8-8 (1.1 g) was prepared by PHPLC (CH_3_CN–H_2_O (16:84, *v*/*v*) + 1% HOAc), and (7*S*,8*R*)-aegineoside (**20**, 10.1 mg) was yielded. Fraction 1-8-10 (917.9 mg) was purified by PHPLC (CH_3_CN–H_2_O (13:87, *v*/*v*) + 1% HOAc) to obtain astragalin (**4**, 4.6 mg) and isorhamnetin 3-*O*-β-d-glucopyranoside (**9**, 21.3 mg). Fraction 1-8-11 (1.3 g) was isolated by PHPLC (CH_3_CN–H_2_O (25:75, *v*/*v*)) to give 1(*E*),2(*E*)-di-*O*-sinapoyl β-d-glucopyranoside (**17**, 414.4 mg). Fraction 1-12 (8.0 g) was subjected to ODS CC (MeOH–H_2_O (10% → 20% → 30% → 40% → 50% → 70% → 100%, *v*/*v*)), and nine fractions (Fr. 1-12-1–1-12-9) were given. Fraction 1-12-8 (1.4 g) was further prepared by PHPLC (CH_3_CN–H_2_O (14:86, *v*/*v*) + 1% HOAc) to yield sinapoyl-9-sucrosecoside (**16**, 370.6 mg). Fraction 1-12-9 (2.1 g) was purified by PHPLC (MeOH–H_2_O (40:60, *v*/*v*) + 1% HOAc) to obtain 1,2-disinapoylgentiobiose (**18**, 1.3 g) and drabanemoroside (**6**, 57.3 mg). Fraction 1-13 (13.7 g) was isolated by PHPLC (MeOH–H_2_O (15:85 → 30:70 → 38:62 → 48:52, *v*/*v*) → MeOH) to give twenty-one fractions (Fr. 1-13-1–1-13-21). Fraction 1-13-3 (606.1 mg) was purified by PHPLC (CH_3_CN–H_2_O (5:95, *v*/*v*) + 1% HOAc) to gain apetalumoside D (**2**, 120.0 mg). Fraction 1-13-4 (780.2 mg) was separated by PHPLC (CH_3_CN–H_2_O (8:92, *v*/*v*) + 1% HOAc) to yield l-tryptophan (**21**, 102.4 mg). Fraction 1-13-6 (543.7 mg) was further purified by PHPLC (CH_3_CN–H_2_O (8:92, *v*/*v*)) to obtain 4,9-di-*O*-β-d-glucosyl sinapoyl alcohol (**13**, 32.0 mg). Fraction 1-13-16 (217.6 mg) was isolated by PHPLC (CH_3_CN–H_2_O (16:84, *v*/*v*)), and isorhamnetin 3,4′-*O*-β-d-diglucoside (**10**, 35.2 mg) was yielded. Fraction 1-13-17 (369.6 mg) was prepared by PHPLC (CH_3_CN–H_2_O (18:82, *v*/*v*)) to gain apetalumoside C_1_ (**1**, 54.1 mg). Fraction 1-13-20 (779.2 mg) was separated by PHPLC (CH_3_CN–H_2_O (16:84, *v*/*v*)) to obtain quercetin 3-*O*-α-l-rhamnopyranosyl(1→2)-α-l-arabinopyranoside (**8**, 273.6 mg). Fraction 1-14 (8.0 g) was subjected to Sephadex LH-20 CC (MeOH–H_2_O (1:1, *v*/*v*)), and seven fractions (Fr. 1-14-1–1-14-7) were given. Fraction 1-14-7 (477.8 mg) was separated by PHPLC (CH_3_CN–H_2_O (14:86, *v*/*v*) + 1% HOAc) to yield 2-*O*-(3,4-dihydroxybenzoyl)-2,4,6-trihydroxyphenylacetic acid 4-*O*-β-d-glucopyranoside (**12**, 6.2 mg). Fraction 1-15 (14.1 g) was isolated by PHPLC (CH_3_CN–H_2_O (9:91, *v*/*v*)), and eleven fractions (Fr. 1-15-1–1-15-11) were obtained. Fraction 1-15-1 (2.5 g) was further prepared by PHPLC (CH_3_CN–H_2_O (8:92, *v*/*v*)) to give quercetin 3-*O*-β-d-glucopyranosyl-7-*O*-β-d-gentiobioside (**7**, 265.6 mg). Fraction 1-15-5 (2.3 g) was subjected to Sephadex LH-20 CC (MeOH–H_2_O (1:1, *v*/*v*)) and finally separated by PHPLC (CH_3_CN–H_2_O (10:90, *v*/*v*)) to yield kaempferol 3-*O*-β-d-glucopyranosyl-7-*O*-β-d-gentiobioside (**5**, 197.1 mg). Fraction 1-15-8 (294.4 mg) was purified by PHPLC (CH_3_CN–H_2_O (9:91, *v*/*v*)) to gain isorhamnetin 3-*O*-β-d-glucopyranosyl-7-*O*-β-d-gentiobioside (**11**, 140.5 mg).

Meanwhile, fraction 2 (4.0 g) was isolated by PHPLC (MeOH–H_2_O (2:98, *v*/*v*)), and seven fractions (Fr. 2-1–2-7) were given. Fractions 2-4 (102.8 mg) and 2-5 (159.6 mg) were further purified by PHPLC (MeOH–H_2_O (1:99, *v*/*v*)) to yield stachyose (**24**, 40.9 mg) and 1-thio-β-d-glucopyranosyl(1→1)-1-thio-α-d-glucopyranoside (**3**, 73.3 mg). Fraction 2-6 (102.8 mg) was separated by PHPLC (MeOH–H_2_O (3:97, *v*/*v*) to gain TgSSTg (**25**, 58.3 mg).

*Apetalumoside C_1_* (**1**): Yellow powder; [*α*]${}_{D}^{25}$ −41.1° (*c* = 0.95, MeOH); IR *υ*_max_ (KBr) cm^−1^: 3362, 2937, 1699, 1653, 1600, 1516, 1457, 1340, 1286, 1179, 1113, 1066, 827; UV *λ*_max_ (MeOH) nm (log *ε*): 334 (4.19), 266 (4.09), 245 (4.20). ^1^H- (DMSO-*d*_6_, 500 MHz) and ^13^C-NMR (DMSO-*d*_6_, 125 MHz) spectroscopic data, see Table 1. HRESI–TOF–MS: Negative-ion mode *m*/*z* 977.2555 [M − H]^−^ (calcd. for C_44_H_49_O_25_, 977.2568).

*Apetalumoside D* (**2**): White powder; [*α*]${}_{D}^{25}$ −35.3° (*c* = 0.94, MeOH); IR *υ*_max_ (KBr) cm^−1^: 3399, 2922, 1616, 1519, 1463, 1336, 1222, 1113, 1025, 876, 825; UV *λ*_max_ (MeOH) nm (log *ε*): 277 (3.28, sh); 242 (3.82). ^1^H- (DMSO-*d*_6_, 500 MHz) and ^13^C-NMR (DMSO-*d*_6_, 125 MHz) spectroscopic data, see Table 2. HRESI–TOF–MS: Positive-ion mode *m*/*z* 593.1333 [M + Na]^+^ (calcd. for C_22_H_34_O_13_S_2_Na, 593.1333).

*1-Thio-β-d-glucopyranosyl(1→1)-1-thio-α-d-glucopyranoside* (**3**): White powder. [*α*]${}_{D}^{25}$ +184.5° (*c* = 0.97, H_2_O); IR *υ*_max_ (KBr) cm^−1^: 3368, 2888, 1636, 1411, 1356, 1273, 1097, 1042, 874; ^1^H- (D_2_O, 500 MHz) and ^13^C-NMR (D_2_O, 125 MHz) spectroscopic data, see Table 3. HRESI–TOF–MS: Positive-ion mode *m*/*z* 391.0739 [M + H]^+^ (calcd. for C_12_H_23_O_10_S_2_, 391.0727).

*Sinapoyl-9-sucrosecoside* (**16**): Pale yellow powders; The NMR data of **16** in DMSO-*d*_6_ is first reported. ^1^H-NMR (DMSO-*d*_6_, 500 MHz) δ: 6.89 (2H, s, H-2,6), 7.60 (1H, d, *J* = 16.0 Hz, H-7), 6.44 (1H, d, *J* = 16.0 Hz, H-8), 3.65 ((1H, d, *J* = 12.5 Hz), 3.69 (1H, d, *J* = 12.5 Hz), H_2_-1′), 3.87 (1H, d, *J* = 10.0, H-3′), 4.12 (1H, dd, *J* = 8.0, 10.0 Hz, H-4′), 4.18 (1H, m, H-5′), (4.32 (1H, dd, *J* = 6.0, 12.0 Hz), 4.55 (1H, br. d, ca. *J* = 12 Hz, H_2_-6′)), 5.46 (1H, d, *J* = 3.0 Hz, H-1′′), 3.54 (1H, dd, *J* = 3.0, 9.5 Hz, H-2′′), 3.82 (1H, dd, *J* = 9.5, 9.5 Hz, H-3′′), 3.41 (1H, dd, *J* = 9.5, 9.5 Hz, H-4′′), 4.18 (1H, m, H-5′′), (3.83 (1H, m, overlapped), 3.92 (1H, br. d, ca. *J* = 11 Hz), H_2_-6′′), 3.87 (6H, s, 3,5-OCH_3_); ^13^C-NMR (DMSO-*d*_6_, 125 MHz) δ: 126.5 (C-1), 106.8 (C-2,6), 149.2 (C-3,5), 139.3 (C-4), 147.3 (C-7), 115.5 (C-8), 169.2 (C-9), 64.1 (C-1′), 105.1 (C-2′), 83.6 (C-3′), 76.0 (C-4′), 79.0 (C-5′), 65.1 (C-6′), 93.1 (C-1′′), 73.0 (C-2′′), 74.5 (C-3′′), 71.7 (C-4′′), 71.9 (C-5′′), 64.1 (C-6′′), 56.9 (3,5-OCH_3_); HRESI–TOF–MS: Negative-ion mode *m*/*z* 547.1680 [M − H]^−^ (calcd. for C_23_H_31_O_15_, 547.1668).

*Acid Hydrolysis of* **1**: the solution of compound **1** (2.0 mg) in 1 M HCl (1 mL) was treated by using the same method as described in reference [1,2]: **1** was heated under reflux for 3 h. The reaction mixture was then analyzed by CH_3_CN–H_2_O (70:30, *v*/*v*; flow rate 1.0 mL/min). As a result, d-glucose was detected from the aqueous phase of **1** by comparison of its retention time and optical rotation with that of the authentic sample, d-glucose (*t*_R_ 8.8 min (positive)).

### 3.4. Evaluation of Effects on Sodium Oleate-Induced TG Overloading in HepG2 Cells

*Materials*: HepG2 cells were purchased from Cell Resource Center of Institute of Basic Medical Sciences, Chinese Academy of Medical Sciences & Peking Union Medical College (Beijing, China). Dulbecco’s modified Eagle’s medium (DMEM), penicillin and streptomycin were purchased from Thermo Scientific (Waltham, MA, USA). Fetal Bovine Serum (FBS) was obtained from Mediatech (Herndon, VA, USA). TG assay kits were purchased from Biosino Bio-Technology And Science Inc. (Beijing, China). Sodium oleate (SO) and orlistat were obtained from Sigma-Aldrich Corporation (St. Louis, MO, USA).

*Cell culture*: HepG2 cells were routinely cultured in DMEM-based medium as described before [25]. After cells reached about 80% confluence and were seeded at a density of 80,000 cells/mL in 48-multiwell plates for 24 h, the experiments were then performed.

*Induction and evaluation of TG overloading*: TG overloading was induced as described before [25]. Briefly, HepG2 cells at 80% confluence were exposed to 200 µmol/L SO for 48 h. Meanwhile, the tested isolates at the indicated concentrations were added in the presence of SO. Orlistat (5 µmol/L) was selected as the positive control and the medium without SO was used as the negative control. At the end of the experiment, the intracellular TG content was determined using a commercial TG assay kit after cells were rinsed by phosphate-buffered saline and lysed. The absorbance was analyzed at 492 nm. Under the selected concentrations in this study, according to pre-tests, no obvious influence was observed on cell viability (data not shown). The measurement was made in triplicate.

### 3.5. Statistical Analysis

Statistical analyses were undertaken with SPSS v12.0 (SPSS, Chicago, IL, USA). The significance of the differences between the mean values was determined using an analysis of variance (ANOVA). The differences were considered statistically significant at *p* < 0.05.

## 4. Conclusions

Summed up, twenty-five compounds (**1**–**25**) including three new ones, apetalumosides C_1_ (**1**), D (**2**), and 1-thio-β-d-glucopyranosyl(1→1)-1-thio-α-d-glucopyranoside (**3**), were obtained from the seeds of *L. apetalum*. Among the known isolates, **5**–**8**, **10**–**13**, **16**–**20**, and **25** were obtained from the genus for the first time; **4**, **14**, **15**, **21**–**24** were isolated from the species for the first time. Meanwhile, the NMR data of **16** was first reported here. Their structures were determined by means of chemical and spectroscopic methods. Moreover, their inhibitory effects on TG overloading were evaluated in HepG2 cells. The results showed that phenol compounds, including five flavonoids (**7**–**11**), five sinapic acid groups (**13**, **14**, **16**–**18**) and one lignin (**20**), together with two new compounds (**1** and **2**) as well as two other isolates (**21** and **25**) have significant TG-lowering effects, among of which, **10**, **13** and **21** exhibited a level of activities almost comparable to that of orlistat. It is suggested that the above compounds contained in the *L. apetalum* might be part of the material basis involved in the lipid metabolism.

## Figures and Tables

**Table 1:** ^1^H- and ^13^C-NMR data for **1** in DMSO-*d*_6_.

No.	δ_C_	δ_H_ (*J* in Hz)	No.	δ_C_	δ_H_ (*J* in Hz)
2	156.7	—	2′′′	73.0	3.28 (dd, 7.0, 9.5)
3	133.4	—	3′′′	76.2	3.32 (dd, 9.5, 9.5)
4	177.6	—	4′′′	69.2	3.26 (m, overlapped)
5	160.8	—	5′′′	75.3	3.75 (m)
6	99.4	6.51 (br. s)	6′′′	68.9	3.71 (dd, 5.5, 11.5)
7	162.7	—			3.99 (br. d, ca. 12)
8	94.4	6.85 (br. s)	1′′′′	103.5	4.35 (d, 8.0)
9	155.9	—	2′′′′	71.5	3.22 (dd, 7.5, 8.0)
10	105.6	—	3′′′′	77.5	4.90 (dd, 7.5, 9.0)
1′	120.7	—	4′′′′	68.1	3.34 (dd, 9.0, 9.0)
2′,6′	130.9	8.09 (d, 9.0)	5′′′′	77.4	3.08 (m)
3′,5′	115.2	6.92 (d, 9.0)	6′′′′	60.7	3.56 (br. d, ca. 12)
4′	160.1	—			3.70 (dd, 5.5, 11.5)
5-OH	—	12.65 (br. s)	1′′′′′	124.5	—
1′′	100.6	5.50 (d, 8.0)	2′′′′′,6′′′′′	105.9	7.00 (s)
2′′	74.2	3.21 (dd, 7.5, 8.0)	3′′′′′,5′′′′′	147.9	—
3′′	76.8	3.26 (m, overlapped)	4′′′′′	138.0	—
4′′	69.8	3.08 (m, overlapped)	7′′′′′	144.9	7.51 (d, 16.0)
5′′	76.3	3.21 (m)	8′′′′′	115.5	6.53 (d, 16.0)
6′′	60.8	3.30 (br. d, ca. 11)	9′′′′′	166.2	—
		3.50 (dd, 5.5, 10.5)	3′′′′′,5′′′′′-OCH_3_	56.0	3.81 (s)
1′′′	99.7	5.12 (d, 7.0)			

**Table 2:** ^1^H- and ^13^C-NMR data for **2** in DMSO-*d*_6_.

No.	δ_C_	δ_H_ (*J* in Hz)	No.	δ_C_	δ_H_ (*J* in Hz)
1	130.2	—	4′	70.0 ^a^	3.07 (m, overlapped)
2,6	105.6	6.58 (s)	5′	78.1	3.12 (m, overlapped)
3,5	147.5	—	6′	61.2 ^b^	3.46 (dd, 5.0, 12.5)
4	134.5	—			3.70 (br. d, ca. 13)
7	46.5	4.28 (br. d, ca. 5)	1′′	84.7	4.27 (d, 10.0)
8	34.8	3.18 (br. d, ca. 11)	2′′	80.8	3.13 (m, overlapped)
		3.39 (dd, 5.0, 11.0)	3′′	78.0	3.12 (m, overlapped)
3,5-OCH_3_	55.9	3.75 (s)	4′′	69.9 ^a^	3.07 (m, overlapped)
1′	83.7	4.31 (d, 9.5)	5′′	72.9	3.01 (dd, 9.5, 10.0)
2′	72.9	3.01 (dd, 8.0, 9.5)	6′′	61.1 ^b^	3.46 (dd, 5.0, 12.5)
3′	80.8	3.13 (m, overlapped)			3.70 (br. d, ca. 13)

^a,b^ Can be exchanged.

**Table 3:** ^1^H- and ^13^C-NMR data for **3** in D_2_O.

No.	δ_C_	δ_H_ (*J* in Hz)	No.	δ_C_	δ_H_ (*J* in Hz)
1	96.1	5.57 (d, 5.5)	1′	92.4	4.68 (d, 9.0)
2	74.3	3.87 (dd, 5.5, 9.5)	2′	74.4	3.49 (dd, 9.0, 9.5)
3	76.2	3.58 (dd, 9.5, 9.5)	3′	80.0	3.52 (dd, 9.5, 9.5)
4	72.3	3.44 (dd, 9.5, 9.5)	4′	72.1	3.42 (dd, 9.5, 9.5)
5	76.4	3.94 (m)	5′	83.1	3.50 (m)
6	63.4	3.80 (dd, 5.5, 12.5)	6′	63.7	3.72 (dd, 5.5, 12.5)
		3.88 (dd, 1.5, 12.5)			3.91 (dd, 1.5, 12.5)

**Table 4:** TG clearance of compounds **7**, **8** and **17** at different concentrations.

Sample (µmol/L)	7			8			17		
	30	3	0.3	30	3	0.3	30	3	0.3
TG clearance (%)	9.46 ± 1.89	7.16 ± 0.87	3.24 ± 0.35	12.00 ± 1.17	8.27 ± 2.14	4.15 ± 1.97	12.70 ± 2.39	5.68 ± 1.15	4.32 ± 1.57

**Table 5:** TG clearance of compounds **20** and **25** at different concentrations.

Sample (µmol/L)	20				25			
	100	30	3	0.3	100	30	3	0.3
TG clearance (%)	4.14 ± 1.40	3.91 ± 1.79	2.82 ± 1.06	1.31 ± 1.02	12.66 ± 0.77	3.91 ± 0.90	2.96 ± 0.45	2.00 ± 0.34
